# ISCB’s initial reaction to *New England Journal of Medicine* editorial on data sharing

**DOI:** 10.1093/bioinformatics/btw090

**Published:** 2016-03-24

**Authors:** Bonnie Berger, Theresa Gaasterland, Thomas Lengauer, Christine A Orengo, Bruno Gaeta, Scott Markel, Alfonso Valencia

**Affiliations:** International Society for Computational Biology, Inc. (ISCB), 9650 Rockville Pike Bethesda, Maryland, USA

The recent editorial by Dr Longo and Dr Drazen in the *New England Journal of Medicine* (Longo and Drazen, 2016) has stirred up quite a bit of controversy. As Executive Officers of the International Society of Computational Biology, Inc. (ISCB), we express our deep concern about the restrictive and potentially damaging opinions voiced in this editorial, and while ISCB works to write a detailed response, we felt it necessary to promptly address the editorial with this reaction. Although some of the concerns voiced by the authors of the editorial are worth considering, large parts of the statement purport an obsolete view of hegemony over data that is neither in line with today’s spirit of open access nor furthering an atmosphere where the potential of data can be fully realized.

ISCB acknowledges that the additional comment on the editorial (Drazen, 2016) eases some of the polemics unfortunately without addressing some of the core issues. We still feel, however, that we need to contrast the opinion voiced in the editorial with what we consider the axioms of our scientific society, statements that lead into a fruitful future of data-driven science:

- Data produced with public money should be public in benefit of the science and society
- Restrictions on the use of public data hamper science and slow progress
- Open data is the best way to combat fraud and misinterpretations

Current large data collections proceed from many sources, are continually accumulated, and require a variety of analytical approaches. Data generation and data analysis overlap in time and are continually updated with new data sets produced by new techniques and new analysis methodologies. Furthermore, in many cases current science functions in consortia in which scientists collaborate toward common goals while preserving their own scientific objectives. Dividing scientists into data providers and data analysts is simplistic and gives a misleading impression of the actual state of biological and biomedical science.

ISCB very much supports collaboration between disciplines, including experimental and clinical as well as bioinformatics, as the best way forward to address complex biological problems. But this collaboration cannot be based on imposed restrictions to data access and cannot be contained in professional silos. (The use of expressions such as ‘research parasites’ clearly does not help.)

Many bio-communities have made significant progress by endorsing open data policies and, gratefully, public funding agencies have connected to the spirit that they are distributing taxpayers’ money to science and that, therefore, the data that are generated in the course belong to the public. It is, perhaps, natural that some areas of biomedical research are slow in adopting these policies. History and the confidential nature of the relevant data are surely among the reasons. However, in our opinion data hegemony is another, a reason that has to be overcome. The sooner these barriers to progress are removed the sooner the patients will benefit from the current flourishing of biomedical research.

*Conflict of Interest*: none declared.
